# Antiplasmodial Activity and Pharmacokinetic Profiling of Cryptolepine and 2,7‐Dibromocryptolepine With a View to Informing the Design of Novel Antimalarial Cryptolepine Analogues

**DOI:** 10.1002/ddr.70245

**Published:** 2026-02-03

**Authors:** Elodie Chenu, James Duffy, Arnold Donkor Forkuo, Stephen Y. Gbedema, Seham Abdelall, Muhammad Wahajuddin, Huw S. Jones, Colin W. Wright

**Affiliations:** ^1^ MMV Medicines for Malaria Venture Meyrin Switzerland; ^2^ Department of Pharmacology Faculty of Pharmacy and Pharmaceutical Sciences, College of Health Sciences Kwame Nkrumah University of Science and Technology (KNUST) Kumasi Ghana; ^3^ Department of Pharmaceutics Faculty of Pharmacy and Pharmaceutical Sciences, College of Health Sciences Kwame Nkrumah University of Science and Technology (KNUST) Kumasi Ghana; ^4^ School of Pharmacy and Medical Sciences University of Bradford Bradford UK; ^5^ Institute of Cancer Therapeutics School of Pharmacy and Medical Sciences University of Bradford Bradford UK

**Keywords:** 2,7‐dibromocryptolepine, antimalarial drug development, cryptolepine, *Cryptolepis sanguinolenta*, enterohepatic circulation, hERG

## Abstract

The roots of the climbing shrub *Cryptolepis sanguinolenta* are traditionally used in West Africa for the treatment of malaria. The principal constituent, cryptolepine (**1**), has been shown to have antimalarial activity but there are concerns regarding its toxicity on account of its DNA‐intercalating property. The synthetic analogue, 2,7‐dibromocryptolepine, (**2**) does not intercalate into DNA and is markedly more active than the parent against *Plasmodium* sp. in vitro and in vivo. The aim of this study was to carry out a pre‐clinical assessment of **1** and **2**, and if appropriate, carry out in vivo pharmacokinetic studies. Cryptolepine (**1**) and 2,7‐dibromocryptolepine (**2**), were evaluated in a range of in vitro assays in line with those recommended by Medicines for Malaria Venture (MMV) for the profiling of a Validated Hit Compound (https://www.mmv.org/frontrunner-templates). In vitro profiling of **1** and **2** showed that **2** is superior to **1** with respect to antiplasmodial activities, and the parasite rate of kill (fast for **2**, in contrast with modest for **1**); however **2** exhibited potent inhibition of the hERG potassium channel, (IC_50_ = 1.0 µM compared with 7.8 µM for **1**), raising concerns that **2** may be cardiotoxic, so that **2** was not selected for in vivo pharmacokinetic profiling. The studies of cryptolepine (**1**) pharmacokinetics in the rat revealed a second peak, especially with oral administration, indicating that enterohepatic circulation following biliary excretion may be taking place. This study complements previous pharmacokinetic data of **1** and presents novel data on 2,7‐dibromocryptolepine (**2**) that will inform the development of cryptolepine analogues as potential antimalarial agents.

**Abbreviations**
DMSO: dimethylsulfoxide
FBS: fetal bovine serum
hERG: human ether‐á‐go‐go‐related gene
IP: intra‐peritoneal
LDH: lactate dehydrogenase
MMV: Medicines for Malaria Venture
PBS: phosphate buffered saline
*Pf*LDH: *P. falciparum* lactate dehydrogenase
*P. falciparum*
: *Plasmodium falciparum*
SE: standard error

## 1 Introduction

**Figure 1:**
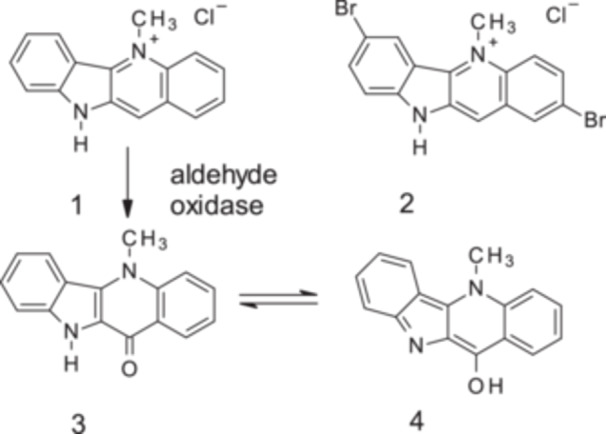
Structures of cryptolepine (**1**), 2,7‐dibromocryptolepine (**2**), cryptolepine‐11‐one (**3**), 11‐hydroxycryptolepine (**4**), and metabolism of **1** by aldehyde oxidase.

The roots of the climbing shrub, *Cryptolepis sanguinolenta* (Lindl.) Schltr. (Apocynaceae) are used in West Africa for the treatment of malaria. In Ghana, an herbal preparation, Phyto‐Laria, is available and has been evaluated in a clinical study (Bugyei et al. 2010). The indoloquinoline alkaloid, cryptolepine, (5‐methyl‐10*H*‐indolo[3,2‐*b*]quinoline) (**1,** Figure 1) is the principal constituent of *C. sanguinolenta* roots and has been shown to have in vitro antiplasmodial activity (IC_50_ against chloroquine‐resistant *Plasmodium falciparum* strain K1 = 0.44 µM), as well as oral activity against *P. berghei* in mice (80% suppression of parasitaemia at 50 mg/kg) (Wright et al. 1996). In addition, **1** has been shown to be active against late‐stage (IV/V) gametocytes of *P. falciparum* (IC_50_ = 1.97 µM) (Forkuo, Ansah, Mensah, et al. 2017). However, **1** does not cure malaria in mouse in vivo studies and concerns have been expressed regarding its toxicity.

Cryptolepine (**1**), has been shown to be moderately cytotoxic to human cancer cells and the mechanisms involved include intercalation into DNA, inhibition of DNA synthesis, inhibition of topoisomerase II, and induction of apoptosis (Dassonneville et al. 2000). A crystal structure of **1** intercalated into short DNA sequences revealed a previously unknown mode of intercalation in which cryptolepine preferentially intercalates between non‐alternating GC sites (Lisgarten et al. 2002). As a consequence of its DNA intercalating ability, **1** may be potentially genotoxic, although studies have shown DNA damage only at relatively high (300 µM) concentrations (Ansah et al. 2005; Gopalan et al. 2011). In addition, ex vivo studies in zebrafish embryos found that **1** induced significant developmental malformations, growth retardation, and mortality at 100 µM but not at lower concentrations (Mensah et al. 2019). Although the pharmacokinetics of cryptolepine have been previously explored in the rat (Forkuo, Ansah, Pearson, et al. 2017), the observation time was short. In light of the toxicity of **1**, a longer assessment of in vivo exposure is needed to better understand the in vitro data and, where appropriate, determine safety margins.

In an attempt to reduce toxicity and increase antimalarial potency through structural modification, a number of synthetic analogues of **1** have been prepared including dihalogenated analogues that were found to be up to 10‐fold more potent than **1** against chloroquine‐resistant *P. falciparum* (strain K1) in vitro and suppressed parasitaemia in *P. berghei‐*infected mice by 90% at 25 mg/kg/day I.P. (Onyeibor et al. 2005). The best studied dihalogenated analogue, 2,7‐dibromocryptolepine (**2**, Figure 1), was not toxic to mice in contrast to **1** which killed the mice at 25 mg/kg I.P., and in addition, **2** has been shown not to intercalate into DNA (Lisgarten et al. 2002). In addition, in contrast to **1**, the 2,7‐dibromo‐analog **2** is not inactivated by liver aldehyde oxidase (Figure 1, Stell et al. 2012), which may contribute to its relatively high antimalarial potency in vivo. As with **1**, the antiplasmodial mode of action of **2** involves the inhibition of haemozoin formation, but this does not fully account for the increased antiplasmodial potency of **2** suggesting that additional, unknown mechanism(s) of action are operating, and this may also explain why **2** has been found to be resilient to *P. falciparum* resistance development (Abacha et al. 2022).

These findings indicate that further work contextualizing the reported in vitro toxicity of **1** and of analogues of **1** as leads to novel antimalarial drugs would be worthwhile in order to inform the design of new analogues. To address these existing limitations, this study reports the profiling of **1** and **2** against the MMV criteria for Validated Hit Compounds (https://www.mmv.org/frontrunner-templates), to determine whether **2** may be suitable for optimization to a novel antimalarial “Early Lead” with potential for further development toward a drug candidate. The data obtained for **1** complements previous work, provides “base‐line” data with which the profile of **2** will be compared and will also be valuable for **1** on account of the use of the source plant, *C. sanguinolenta* in West African traditional medicine.

## 2 Materials and Methods

Except where otherwise stated, the data presented in this study were obtained through MMV by arrangement with TCG Lifesciences Pvt. Ltd., Registered Office, Block BN, Plot 7, Sector V, Salt Lake Electronics Complex, Kolkata, 700 091, West Bengal, India.

### 2.1 Materials

The sun‐dried, cut roots of *C. sanguinolenta* used in this study were obtained from the Centre for Plant Medicine Research (formerly the Centre for Scientific Research into Plant Medicine), Mampong‐Akwapim, Ghana, in August 2012 and were identified at the Plant Development Centre of the Institution. Their authenticity was confirmed by Dr G. H. Sam of the KNUST Herbal Medicine Department by comparison with a voucher specimen KNUST/HMI/2008/L056 at the KNUST Herbal Medicine Department herbarium in Kumasi, Ghana. Cryptolepine (**1**) was extracted and isolated from the roots as previously described (Abacha et al. 2022). 2,7‐Dibromocryptolepine, (**2**), was synthesized as previously described (Wright et al. 2001).

### 2.2 In Vitro *P. falciparum* Viability Assay

*P. falciparum* (3D7 and Dd2 strains, obtained from BEI Resources (https://www.beiresources.org/Home.aspx) were cultured in human red blood cells with RPMI‐1640 (10.3 g/L), supplemented with l‐glutamine (2 mM), hypoxanthine (150 µM), HEPES (25 mM), d‐glucose (12 mM), sodium bicarbonate (25.70 mM), and Albumax II (0.5%).

The *Pf*LDH‐based viability assay was performed based on the method described previously (Gamo et al. 2010). Inoculum was prepared from an asynchronous culture (5%−7% parasitemia with ≥ 80% rings); 22.5 µL of parasite inoculum (2% hematocrit, 0.25% parasitemia) in complete medium containing 0.5% Albumax II was added to each well of 384‐well polypropylene plates already containing 2.5 µL of appropriate concentrations of compound in DMSO, mixed well and incubated at 37°C for 72 h in 5% CO_2_, 5% O_2_, 90% N_2._

Following incubation for 72 h, plates were frozen (−80°C, 12 h), and then allowed to thaw (21°C, 5 h). To evaluate *Pf*LDH activity, 70 µL of freshly prepared reaction mix [containing lithium l‐lactate (143 mM), 3‐acetyl pyridine adenine dinucleotide (APAD) (143 µM), nitro blue tetrazolium chloride (NBT) (178.75 µM), diaphorase (2.83 U/mL), 0.7% Tween 20, and Tris‐HCl pH 8.0 (100 mM)] was added to each well. Plates were incubated in the dark for 20 min at 21°C and absorbance at 650 nm was recorded in a plate reader (Spectramax M5). Data was analyzed using standard methodology. Chloroquine diphosphate and atovaquone were used as reference standards for 3D7 assays, and atovaquone and dihydroartemisinin for Dd2 assays.

### 2.3 MMV Library Design Antimalarial Scoring Profile

This enables the prioritization of compounds. It uses six properties including alerts for undesirable substructures and common “drug‐like” properties. The maximum score is 1, and the value gives a measure of the overall property profile weighted to key properties (https://optibrium.com/resources/mmv-antimalarial-scoring-profile/).

### 2.4 In Vitro Cytotoxicity Assay With HepG2 Cells

HepG2 cells were cultured in DMEM supplemented with sodium pyruvate (1 mM), HEPES (10 mM), and 10% fetal bovine serum (FBS). HepG2 cells were sub‐cultured in growth medium (DMEM + 10% FBS) 48 h prior to cell plating. Cells were plated in 384‐well clear‐bottom culture plates with a cell count of 2000 cells per well in 50 µL of medium and incubated for 24 h in a CO_2_ incubator so that 30%−40% of confluency was obtained on the day of treatment with test compounds. After 24 h of incubation, medium was discarded carefully and 45 µL of fresh medium was added to each well. Cells were treated with either 5 µL of vehicle (5% DMSO) or the desired concentration of the test compound and incubated at 37°C for 72 h. The final DMSO concentration in the cell plate was 0.5%. In the positive control wells (100% inhibition), cells were treated with 5 µL of 1% Triton (final assay conc. 0.1%). Following incubation for 72 h, 25 µL of medium was discarded from each well and 25 µL of Cell Titer Glo reagent was added. Following the addition of reagent, the plate was incubated for 15 min at 25°C in a thermomixer with shaking (300 rpm) to develop the luminescent signal. Luminescence was measured (Spectramax M5), and the data were analyzed using standard methodology. Doxorubicin was used as the reference standard.

### 2.5 Albumax Binding

The Albumax binding assay was performed following equilibrium dialysis using RED device inserts. RPMI medium with and without 0.5% Albumax was prepared, and the pH was adjusted to 7.40. 5% Albumax in RPMI medium was spiked with test compound to a 1 µg/mL final concentration, and 300 µL of this was added into the red chamber; 500 µL RPMI medium was added into the white chamber, and the dialysis was set up for 6 h in a modular incubator at 37°C containing 5% CO_2_ and 95% relative humidity. Post‐incubation samples were taken from both chambers. Biomatrix matching was done for Albumax, RPMI, and initial donor samples, followed by protein precipitation using acetonitrile containing system suitability standard. Plates were shaken at 1000 rpm for 10 min followed by centrifugation at 4200 rpm, 20°C for 20 min. The supernatant was diluted in water containing 30% acetonitrile. Samples were analyzed using LC‐MS/MS. Average % Binding and % Recovery were measured using standard methodology. All samples were prepared in duplicate; ranitidine, propranolol, and warfarin were used as reference standards.

### 2.6 Caco‐2 Permeability

The Caco‐2 permeability study was performed in both A > B and B > A directions. Cells were seeded at a density of 18,750 cells/well and grown for 10 days before assay initiation. The assay was initiated by placing a donor chamber containing 2 µM test compound (prepared in Hanks balanced salt solution containing 10 mM HEPES, pH 7.4) on an acceptor chamber containing the same buffer. Following 2.5 h incubation at 37°C, 5% CO_2,_ and 95% relative humidity, the donor and acceptor chambers were separated, and samples were taken from both sides. Donor, acceptor, and initial donor samples were diluted and quantified using LC‐MS/MS. Membrane integrity was checked using the lucifer yellow test: 0.1 mg/mL lucifer yellow solution was added onto the cell plates post actual study and kept with acceptor assembly at 37°C, 5% CO_2,_ and 95% relative humidity for 1 h. Fluorescence (Ex: 432 nm, Em: 530 nm) was measured from basal wells, after adding 100 µL buffer containing lucifer yellow, 0.1 mg/mL. Wells having more than 1% fluorescence intensity with respect to 0.1 mg/mL lucifer yellow in buffer were considered to have non‐integral membranes. Such wells, if any, were not considered for permeability calculation. Apparent permeability (Papp) value, Efflux ratios, and % Recovery were measured using standard methodology. All samples were prepared in duplicate. Furosemide, atenolol, verapamil, carbamazepine, and domperidone were used as reference standards in the study.

### 2.7 Kinetic Solubility in Phosphate Buffered Saline (PBS) pH 7.4

The test compound (20 mM in DMSO) was added to PBS to obtain a final concentration of 200 µM containing 1% DMSO. Samples were incubated at 25°C with constant shaking (600 rpm) for 2 h, before being filtered using a multiscreen solubility filter plate. The filtrate was diluted 1:1 (v/v) in acetonitrile. A five‐point calibration curve was prepared in PBS:acetonitrile (1:1, v/v) at 200, 150, 75, 25, and 2.5 µM. Blank, calibration, and test samples (*n* = 2) were transferred to a UV‐readable plate, and the plate was scanned for absorbance (200−600 nm). Best‐fit calibration curves were constructed to determine the test sample solubility. The experiment was carried out in duplicate. Diethylstilbestrol, haloperidol, and sodium diclofenac were used as reference standards.

### 2.8 Lipophilicity (LogD_7.4_)

The LogD_7.4_ assay was performed using a miniaturized shake‐flask method. The test compound (10 mM in DMSO) was added to pre‐saturated 1‐octanol layered over PBS (1:1, v/v) to obtain a final overall concentration of 75 µM. Samples were incubated at 25°C with constant shaking (850 rpm) for 2 h. The organic and aqueous phases were then separated by centrifugation (2000 rpm) for 10 min at 25°C, and samples of each phase transferred to plates for dilution. The organic phase was diluted to 1000‐fold with a water‐methanol mixture (10:90, v/v) containing an internal standard, and the aqueous phase was diluted 20‐fold with a water‐methanol mixture (10:90, v/v) containing an internal standard. The relative concentrations in the two phases were determined using LC‐MS/MS. The experiment was carried out in duplicate. Propranolol, amitriptyline, and midazolam were used as reference standards.

### 2.9 hERG Potassium Channel Inhibition

This was carried out by B'SYS GmbH, Switzerland (http://www.bsys.ch) using automated patch‐clamping of hERG potassium channels expressed in CHO cells. hERG currents were recorded from stably transfected CHO cells (hERG DUO, B'SYS GmbH), using automated patch‐clamping (Q‐Patch, Sophion). Cells were cultivated under standard conditions and passaged at a confluence of 50% to 80%. Extracellular solution for electrophysiological experiments contained (in mM) 137 NaCl, 4 KCl, 1.8 CaCl_2_, 1 MgCl_2_, 10 HEPES, 10 d‐Glucose, pH (NaOH) 7.4, intracellular solution contained (in mM) 130 KCl, 2 CaCl_2_, 4 MgCl_2_, 4 Na_2_ATP, 10 HEPES, 5 EGTA, pH (KOH) 7.2. After forming the GΩ seal and whole cell configuration, cells were clamped to −80 mV and depolarized to +20 mV for 2 s, followed by a voltage step to −40 mV for 3 s, frequency: 0.1 Hz, sampling frequency: 1 kHz. The tail current amplitudes were analyzed. Increasing concentrations of the test item were perfused for at least 250 s per concentration. The steady state current amplitude in the presence of the test item was analyzed and normalized to the initial current amplitude of the same cell in duplicates. Normalized and averaged current amplitudes were fitted with a logistic equation to determine IC_50_ and Hill coefficient.

### 2.10 Metabolic Stability Study Using Human Liver Microsomes

A solution of the test compound in phosphate buffer solution (1 µM) was incubated with pooled human liver microsomes (0.5 mg/mL) for 0, 5, 20, 30, 45, and 60 min at 37°C in the presence of NADPH regeneration system (final DMSO content = 0.005%). The reaction was terminated with the addition of ice‐cold acetonitrile containing an internal standard (final acetonitrile content = 0.995%). Samples were centrifuged (4200 rpm) for 20 min at 20°C, and the supernatants diluted with water (1:1, v/v), before being analyzed by LC‐MS/MS. The % compound remaining, half‐life (T_1/2_), and intrinsic clearance (CL_int_) were calculated using standard methodology. The experiment was carried out in duplicate. Verapamil, diltiazem, phenacetin, and imipramine were used as reference standards.

### 2.11 Metabolic Stability Study Using Cryopreserved Rat Hepatocytes

A solution of the test compound (1 µM) in Krebs−Henseleit buffer was incubated with pooled rat hepatocytes (1 × 10^6^ cells/mL) for 0, 15, 30, 45, 60, 75, and 90 min at 37°C (5% CO_2_, 95% relative humidity). The reaction was terminated by the addition of ice‐cold acetonitrile containing an internal standard. Samples were centrifuged (4200 rpm) for 20 min at 20°C, and the supernatants diluted with water (1:1, v/v) before being analyzed by LC‐MS/MS. The % parent compound remaining, half‐life (T_1/2_), and intrinsic clearance (CL_int_) were calculated using standard methodology. The experiment was carried out in duplicate. Diltiazem, 7‐ethoxycoumarin, propranolol, and midazolam were used as reference standards.

### 2.12 Rate of Kill Assay

Parasite killing kinetics were determined as previously described (Linares et al. 2015).

### 2.13 Cytochrome P_450_ Isoform (1A2, 2C9, 2C19, 2D6, 3A4) Inhibition

LC–MS/MS methods were developed on an AB Sciex API4000 (or higher) mass spectrometer integrated with a Shimadzu LC system and a CTC‐PAL autosampler. Parent (Q1) and product (Q3) ions, declustering potential (DP), and collision energy (CE) were optimized using DiscoveryQuant‐assisted analysis, with other parameters set to default values. Chromatographic separation was achieved within 1.2 min using a gradient elution from water with 0.1% formic acid to 80:20 acetonitrile:water containing 0.1% formic acid. Manual tuning of mass and minor LC adjustments were made where necessary. All methods were evaluated for sensitivity, linearity, accuracy, and precision across the relevant detection range, confirming their suitability for quantitation of the test compounds.

Test compounds in 0.1 M phosphate buffer (10 µM) were incubated with pooled human liver microsomes (0.1 mg/mL) for 10 min at 37°C in the presence of an NADPH regeneration system (NRS), containing a cocktail of substrates (2 μM tacrine [1A2], 8 μM diclofenac [2C9], 2 μM dextromethorphan [2D6], and 2 μM midazolam [3A4]). The reaction was terminated with the addition of an equal volume of ice‐cold acetonitrile. Samples were centrifuged (3500 rpm) for 20 min at 15°C and supernatants were analyzed by means of LC‐MS/MS for the presence of specific metabolites of the model substrates. Percent inhibition was calculated based on the reduction in LC‐MS/MS response (peak area) in the presence and absence (solvent control) of the test compound. CYP2C19 inhibition was determined separately following a similar protocol using human liver microsomes (0.2 mg/mL) and 80 µM *S*‐mephenytoin as substrate, with a 20 min incubation. All the experiments were carried out in duplicate. Miconazole was used as a reference standard for all CYPs.

### 2.14 Cryptolepine Pharmacokinetics in the Rat

Sprague Dawley rats (250 ± 50 g; *n* = 3) were used to determine oral and i.v. pharmacokinetic profiles following administration of **1** (1 mg/kg, i.v.) and (3 mg/kg, p.o.). All procedures were carried out subject to TCG Lifesciences Pvt. Kolkata, India Ltd., Institutional Animal Ethics Committee (IAEC) approvals.

## 3 Results and Discussion

### 3.1 In Vitro Profiles of Cryptolepine (1) and 2,7‐Dibromocryptolepine (2)

In previous work (Wright et al. 2001), the antiplasmodial activity of **2** against *P. falciparum* strain K1 was shown to be 10‐fold greater than that of **1** and this has been confirmed in this study against strains 3D7 and Dd2 of *P. falciparum* (Table 1). In addition, in this study, at 10 × IC_50,_ **2** was more effective than **1** in terms of the survival of *P. falciparum* 3D7 (7% survival after 24 h with **2** compared with 27% survival for **1**); this was further confirmed by the rate of kill assay that indicated that the rate of kill of **2** is “fast” (similar to chloroquine) compared with “moderate” for **1** (similar to pyrimethamine) (Table 1). Regarding physicochemical parameters, marked differences between **1** and **2** (as hydrochloride salts) were observed (Table 1), in terms of solubility at pH 7.4 (181 and 9.0 µM), lipophilicity (log D 0.22 and 2.0), and protein binding (Albumax 8% and 36%; human plasma, 62% and 94%), respectively. Studies in Caco‐2 cells (Table 1) indicate that permeability is reasonable despite a small degree of efflux, suggesting that the oral absorption of **1** and **2** is adequate. The intrinsic clearance in human liver microsomes was identical for **1** and **2**, but the intrinsic clearance in rat hepatocytes of **2** was found to be 10‐fold faster than that of **1** with a correspondingly shorter t_1/2_. The dibromo‐analogue (**2**) was found to be an inhibitor of the hERG K^+^ channel (IC_50_ = 1 µM) comparable to its toxicity against HEPG2 cells (IC_50_ = 0.72 µM, SI = 1.3), whereas **1** is more selective (SI = 9.8), but the data indicates a risk of cardiac toxicity for both, potentially resulting in cardiac arrhythmias, especially for **2** (Table 1). The MMV Library Design antimalarial scoring profile (a multiparameter optimization tool with a maximum score of 1), gave scores of 0.72 and 0.46 for **1** and **2**, respectively indicating that **2** has less favorable “drug‐like” properties than **1**, principally due to its high lipophilicity (predicted log P = 5.7). The low profiling score coupled with the potent hERG K^+^ channel inhibition activity of **2** contributed to the decision not to carry out in vivo pharmacokinetic studies with this analogue. Unfortunately, **2** is not suitable for clinical development as an antimalarial drug, but structural modifications leading to a higher profiling score and reduced hERG K^+^ channel inhibition may be feasible. It has been observed that generally, compounds with higher lipophilicity are more likely to interact with hERG, and reducing this parameter may be a successful strategy to reduce hERG activity; similarly, lowering basicity may also be an effective strategy (Garrido et al. 2020), and both are clearly applicable to **2**.

**Table 1:** Antiplasmodial profiles and in vitro pharmacokinetic data for 1 and 2.

Parameter	Cryptolepine HCl (1)	2,7‐Dibromocryptolepine HCl (2)
*P. falciparum* IC_50_, 3D7, 48 h, ^3^H‐hypoxanthine assay	0.27 µM	0.024 µM
*P. falciparum* mean of IC_50_ versus 3D7 and Dd2 strains, both 72 h, LDH assay	1.47 µM	0.027 µM
*P. falciparum* 3D7, % survival at 10 × [IC_50_] at t = 24 and 48 h	27% (24 h); 10% (48 h)	7% (24 h); 7% (48 h)
Rate of kill assay	Moderate	Fast
MMV library design antimalarial scoring profile	0.72	0.46
Kinetic solubility, PBS, 25°C, pH 7.4	181 ± 1.42 µM	9 ± 0.58 µM
Lipophilicity, Log D_7.4_	0.2 ± 0.03	2.0 ± 0.02
Protein binding, Albumax	8 ± 0.9%	36 ± 4.72%
Protein binding, human plasma	62 ± 1.92%	94 ± 0.27%
CACO‐2 permeability, pH 7.4/7.4:		
Apparent permeability (Papp A to B)	3.6 × 10^−6 ^cm/s	1.9 × 10^−6 ^cm/s
Apparent permeability (Papp B to A)	7.1 × 10^−6 ^cm/s	4.0 × 10^−6 ^cm/s
Efflux ratio	2.0	2.2
Intrinsic clearance (human liver microsomes)	16 µL/min/mg; t_1/2_ = 86 min; *n* = 1; slope SE = 0.008; Students *t*‐test, *t* = 2.228; 95% CI	16 µL/min/mg; t_1/2_ = 85 min; *n* = 1; slope SE = 0.006; Students *t*‐test, *t* = 2.228; 95% CI
Intrinsic clearance (rat hepatocytes)	4 µL/min/10^6^ cells; t_1/2_ = 197 min; *n* = 1; slope SE, 0.0002; Students *t*‐test, t = 2.179; 95% CI	43 µL/min/10^6^ cells; t_1/2_ = 16 min; *n* = 1; slope SE, 0.0018; Students *t*‐test, *t* = 2.228, 95% CI
In vitro cytotoxicity, HEPG2 cells, 72 h incubation, IC_50_,	0.80 µM	0.72 µM
Inhibition of hERG, K^+^ channel, IC_50_ (selectivity index with respect to cytotoxicity against HepG2 cells, 72 h)	7.8 µM (9.75)	1.0 µM (1.34)

### 3.2 Inhibition of Cytochrome P_450_ Isoforms

The results of the in vitro studies to determine whether **1** and/or **2** interact with a selection of CYP450 isoforms important in drug metabolism are shown in Table 2. The data suggest that **1** and **2** are potent inhibitors of CYP1A2 or are competing with the substrate, tacrine (IC_50_ ~1 µM), which is not surprising as CYP1A2 substrates are often reported to be planar amides or amines with logD = 0.1−3.6 (Lewis 2002), consistent with the pharmacophores of **1** and **2**. Cryptolepine (**1**), as well as **2** were also found to interact with the metabolism of diclofenac by CYP2D6, but to a lesser extent. In previous work (Stell et al. 2012), has shown that **1** is a substrate for liver aldehyde oxidase resulting in the formation of cryptolepine‐11‐one (**3**, Figure 1), which is tautomeric with 11‐hydroxycryptolepine (**4**, Figure 1, Fort et al. 1998); the latter has been shown to be a minor constituent of *C. sanguinolenta* and was previously reported to have similar activity as **1** against *P. falciparum* KI (IC_50_ = 0.16 and 0.23 µM for **4** and **1**, respectively) (Cimanga et al. 1997). However, Stell et al. (2012), reported that **3** was inactive, and it has now been confirmed in this study that **3** has negligible antiplasmodial activity (*P. falciparum* strain K1, IC_50_ = 20.1 ± 4.11 µM, *n* = 3). In another study, nine metabolites were detected by LC‐MS‐MS following incubation of human and rat hepatocytes with **1** and in the plasma or urine in rats treated orally with **1** (Forkuo, Ansah, Pearson, et al. 2017). Metabolite formation involved hydroxylation and/or glucuronidation, although the substitution patterns were not determined; unchanged cryptolepine detected in urine was negligible.

**Table 2:** Inhibition of CYP_450_ isoforms by cryptolepine and 2,7‐dibromocryptolepine.

Isozyme	Substrate	Cryptolepine (1) % inhibition		2,7‐Dibromocryptolepine (2) % inhibition	
		1 µM	10 µM	1 µM	10 µM
CYP1A2	Tacrine	55.6	93.1	37.8	83.4
CYP2C19	*S*‐Mephenytoin	0	0	12.2	35.3
CYP2D6	Diclofenac	7.6	39.5	20.0	81.8
CYP2C9	Dextromethorphan	0	0	0	0
CYP3A4	Midazolam	0	26.6	0	14.4

### 3.3 Cryptolepine Pharmacokinetics in the Rat

**Figure 2:**
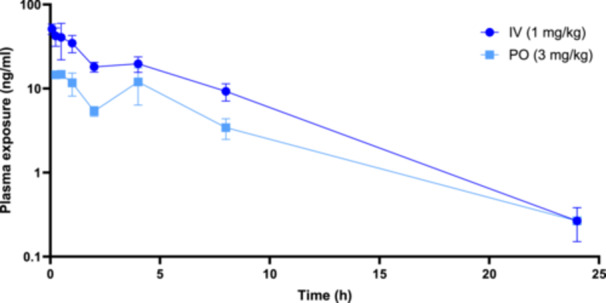
Plasma clearance of cryptolepine (1), in rats following a single IV injection (1 mg/kg) and following a single oral dose (3 mg/kg), *n* = 3.

The plasma concentrations of **1** obtained following the i.v. and oral administration of cryptolepine (**1**) to rats with respect to time are shown in Figure 2 and pharmacokinetic parameters are summarized in Table 3.

**Table 3:** Summary of pharmacokinetic data for cryptolepine (1) following single doses (*n* = 4) by i.v. and oral administration in rats.

Parameter	Intravenous (1 mg/kg)	Oral (3 mg/kg)
Elimination rate constant (Kel)	0.22 L/h	0.19 L/h
Half‐life (T_1/2)_	3.24 h	3.85 h
Maximum concentration (C_max_)	0.06 µg/mL	0.02 µg/mL
Clearance (Cl_0bs)	69.51 mL/min/kg	518.59 mL/min/kg
Mean residence time (MRT)	4.65 h	—
Volume of distribution (Vss)	19.91 L/kg	—
Bioavailability (%BA_0−24_), (F)	—	13.66%

Following oral administration of **1** to rats (3 mg/kg), plasma levels steadily decline for 2 h but then rise again resulting in a second peak at 4 h (Figure 2). The second peak could possibly be due to phases of drug absorption, but a second peak was also observed with i.v. administration, this suggests that enterohepatic recirculation, following biliary secretion, may be the cause. This profile differs from that published in a previous study by Forkuo, Ansah, Pearson, et al. (2017), in which no second peak was observed, possibly because the latter study was shorter (7 h in contrast to 24 h for the study reported here). In addition, the findings that hydroxylated and glucuronidated metabolites have been detected in human and rat hepatocytes incubated with **1** as well as in the plasma and urine of rats administered **1** orally (Forkuo, Ansah, Pearson, et al. 2017), provide further support for the enterohepatic circulation of **1**. The volume of distribution (Vss) of **1** is very high (19.9 L/kg, Table 3), consistent with Vss values observed with other lipophilic quaternary amines (Ryrfeldt and Hansson 1971). However, if there is significant enterohepatic circulation, this will also increase the apparent Vss. The half‐life (T_1/2_) in the rat is relatively short following i.v. (3.24 h) and oral administration (3.85 h), Table 3, and is comparable to the previously published value of 4.5 h (1 mg/kg i.v., *n* = 2) (Forkuo, Ansah, Pearson, et al. 2017). The short half‐life is consistent with the high clearance observed, especially with oral administration (519 mL/min/kg), notwithstanding the large volume of distribution. Oral bioavailability is low, (13.7%, Table 3), and similar to the value of 22% reported by Forkuo, Ansah, Pearson, et al. (2017). Cmax is also low (< 20 ng/mL), but poor absorption is unlikely to be responsible for the low bioavailability as the Caco‐2 permeability data suggest reasonable permeability despite a small degree of efflux, and the solubility is good (Table 1). The study by Forkuo, Ansah, Mensah, et al. (2017), found that less than 1% of the dose was excreted unchanged in the urine within 24 h post dosing and, as discussed above, cryptolepine, (**1**) may be extensively metabolized in the liver. The new finding that **1** undergoes enterohepatic cycling may be beneficial in West Africa, where C. sanguinolenta roots are used traditionally as a decoction for the treatment of malaria, as the bioavailability of **1** may be enhanced.

Overall, this study has shown that **2** is superior to **1** with respect to antiplasmodial properties but its higher lipophilicity and potential hERG inhibitory activity are of concern and indicate that the development of cryptolepine analogues substituted with 1 halogen and an alkylamino group in place of the second halogen may be worthwhile, as it has been shown that a number of 11‐aminoalkyl‐cryptolepine analogues have enhanced antiplasmodial activities with improved selectivity in comparison to **1** (Lavrado et al. 2011). In another study by Mudududdla et al. (2018), a series of indolo[3,2‐*b*]quinoline‐11‐carboxamides were evaluated for antiplasmodial activities, but none were more active than cryptolepine, possibly because these compounds lack the 5‐*N‐*methyl group that has been shown to be essential for potent antiplasmodial activity in cryptolepine (Wright et al. 1996). The incorporation of amino groups into cryptolepine analogues may be expected to reduce lipophilicity, ameliorate the risk of hERG inhibition, and increase oral absorption.

## Author Contributions

James Duffy and Elodie Chenu designed the study and liaised with the companies involved. Arnold Donkor Forkuo and Stephen Y. Gbedema provided *C. sanguinolenta* roots. Seham Abdelall carried out antiplasmodial assays. Elodie Chenu, Muhammad Wahajuddin, and Huw S. Jones evaluated the pharmacokinetic data. Colin W. Wright provided the compounds used in this study and prepared the manuscript. All authors reviewed and approved the final manuscript.

## Ethics Statement

All in vivo procedures were carried out subject to TCG Lifesciences Pvt. Kolkata, India Ltd., Institutional Animal Ethics Committee (IAEC) approvals.

## Consent

The authors have nothing to report.

## Conflicts of Interest

The authors declare no conflicts of interest.

## Data Availability

The data sets supporting the conclusions of this article are included within the article.
